# Natural variation of outcrossing in the hermaphroditic nematode *Pristionchus pacificus*

**DOI:** 10.1186/1471-2148-9-75

**Published:** 2009-04-20

**Authors:** Arielle Click, Chandni H Savaliya, Simone Kienle, Matthias Herrmann, Andre Pires-daSilva

**Affiliations:** 1 Biology Department, University of Texas at Arlington, Arlington, Texas 76019, USA; 2 Department of Evolutionary Biology, Max Planck Institute for Developmental Biology, Spemannstrasse 37–39, D-72076 Tübingen, Germany

## Abstract

**Background:**

Evolution of selfing can be associated with an increase in fixation of deleterious mutations, which in certain conditions can lead to species extinction. In nematodes, a few species evolved self-fertilization independently, making them excellent model systems to study the evolutionary consequences of this type of mating system.

**Results:**

Here we determine various parameters that influence outcrossing in the hermaphroditic nematode *Pristionchus pacificus *and compare them to the better known *Caenorhabditis elegans*. These nematode species are distinct in terms of genetic diversity, which could be explained by differences in outcrossing rates. We find that, similarly to *C. elegans*, *P. pacificus *males are generated at low frequencies from self-fertilizing hermaphrodites and are relatively poor mating partners. Furthermore, crosses between different isolates reveal that hybrids have lower brood sizes than the pure strains, which is a sign of outbreeding depression. In contrast to *C. elegans*, *P. pacificus *has lower brood sizes and the male X-bearing sperm is able to outcompete the X-nullo sperm.

**Conclusion:**

The results indicate that there is no evidence of any selection acting very strongly on *P. pacificus *males.

## Background

The types of mating systems organisms use have important implications for discerning various aspects of the biology of organisms, including genetic diversity, genome evolution, sexual dimorphism and sex ratios [1]. It is therefore crucial to understand the mechanisms and factors that influence the transition of one type of mating system into another. The evolution of mating systems is best understood in flowering plants [2,3], mainly because numerous examples of mating types considered transitory are found in these organisms [4-6]. Hermaphroditism (ie, "cosexuals" or "bisexuals") is considered the ancestral mating system, and selection for outcrossing has been proposed as the main selective force responsible for the evolution of dioecy (male/female) [4,7].

Two main transitory mating systems have been recognized: gynodioecy (females/hermaphrodites) and androdioecy (males/hermaphrodites). In plants, gynodioecy is more prevalent than androdioecy, a trend predicted from theory [4,7,8] and confirmed empirically [9]. According to theoretical predictions, hermaphroditic populations can become gynodioecious when a recessive male-sterility gene spreads in the population [4]. Selection for genetically-fixed gynodioecy is thought to occur as an adaptive response to inbreeding depression of selfing hermaphrodites. The gradual accumulation of subsequent mutations that suppress female functions of hermaphrodites can then result in a dioecious population. Androdioecy is predicted to be a rarer transitory mating system because mutants conferring maleness must achieve at least twice as many outcross fertilizations as hermaphrodites [4,5]. Only in very specific conditions, namely large male advantage, low male frequencies and self-incompatibility, is androdioecy expected to be maintained [10].

In animals, contrary to plants, androdioecy is more common than gynodioecy [11]. This may indicate that there are fundamental differences in the evolution of mating systems in animals as compared to plants. In fact, in most cases it seems that that androdioecy is derived from dioecy [12,13]. Very few detailed models specifying intermediate stages between the evolution of dioecy and hermaphroditism have been proposed, although hermaphroditism has arisen multiple times during animal evolution [14,15]. Selection for reproductive assurance has been invoked as the most plausible explanation for the evolution of hermaphroditism from dioecy.

Nematodes are emerging as a promising model system to understand the conditions and consequences of mating type evolution (eg., [16,17]). The ease of culturing them in the laboratory and the availability of genetic tools for a number of species allows experimental manipulation of breeding types [18-20]. The success of *Caenorhabditis elegans *as genetic model system for developmental biology encouraged the adoption of other hermaphroditic nematodes for comparative studies that include the rhabditids *C. briggsae *[21-23]and *Oscheius tipulae *[24,25], and the more distantly related diplogasterid *Pristionchus pacificus *[26-28].

For rhabditids and diplogasterids androdioecious nematodes, hermaphrodites are basically modified females that produce a limited number of sperm. In contrast to plants, hermaphroditic nematodes cannot fertilize other hermaphrodites. Thus, nematode males are the only outcrossing agents, which give them an advantage over males of androdioecious plant species. Previous models [7,8], which assumed that hermaphrodites cross-fertilize, are therefore not applicable to nematodes.

Because males are nonessential for reproduction in *C. elegans*, models have been developed to explain the maintenance of males [18,29,30]. *C. elegans *males are formed either as a result from rare non-disjunction events during hermaphrodite gametogenesis, or as cross-progeny from hermaphrodite/male crosses. Typically, *C. elegans *males are rarely found in nature and are present as 0.1% of the population in laboratory cultures [31,32]. According to theoretical models, high inbreeding depression, high male mating ability, and high a rate of production of males by hermaphrodite self-fertilization are factors required for maintenance of androdioecy in *C. elegans *[18]. Experiments on the *C. elegans *N2, however, a strain that has been kept in the laboratory for many thousand generations, contradict the theoretical expectations for equilibrium frequency of males [18,29]. Furthermore, *C. elegans *isolates derived recently from the wild seem to counterselect males due to outbreeding depression [33], low non-disjunction rates and relatively poor mating ability [32].

The purpose of this study is to characterize life-history traits of *P. pacificus*, a nematode that evolved androdioecy independently. *P. pacificus *and *C. elegans *are cosmopolitan species with overlapping geographic distribution, but different ecological niches. *C. elegans *has been found mostly in decomposing organic matter (compost piles, farmland and garden soil, and various invertebrates close to decomposing matter) [31,34], while *P. pacificus *has been mostly found in soil samples and in association with scarab beetles [35,36].

Here we characterize a set of variables of life history traits that may influence the maintenance of androdioecy in *P. pacificus*, such as inbreeding depression, male mating ability, brood size and the frequency of male production by hermaphrodites.

## Methods

### Strains

The 23 *P. pacificus *strains used in this study were isolated from the wild in the period between 1988 and 2007 and were inbred in the laboratory for at least 10 generations (Table 1). Strains were isolated from soil, unidentified beetles and beetles of the genus *Phyllophaga *and *Exomala *from the geographical locations described in table 1[37-39]. These strains are a subset of strains from around the world to be described in more detail elsewhere (Herrmann, Kienle, Sommer, unpublished).

**Table 1:** *P. pacificus *isolates

Strain	Location	Region	Origin
JU150	Antananarivo, Madagascar	Africa	Soil
RS5202	Pretoria, South Africa	Africa	Soil
RS5205	Pretoria, South Africa	Africa	Beetle
JU723	Guangxi, China	East Asia	Soil
RS5279	Dinghu Park, China	East Asia	Soil
RS106	Augustow, Poland	Europe	Soil
RS5171	Tivat, Montenegro	Europe	Soil
RS5134	Wooster, Ohio	North America	*Phyllophaga*
PS1843	Port Angeles, Washington	North America	Soil
PS312	Pasadena, California	North America	Soil
RS5207	Hukui, Japan	Pacific	*Exomala*
JU482	Hakone, Japan	Pacific	Soil
RS5188	Hiroshima, Japan	Pacific	*Exomala*
RS5194	Hyogo, Japan	Pacific	*Exomala*
RS5212	Osaka, Japan	Pacific	*Exomala*
RS5214	Hukui, Japan	Pacific	*Exomala*
RS5217	Osaka, Japan	Pacific	*Exomala*
JU138	Captain Hook, Hawaii	Pacific	Soil
RS5270	Amboro Park, Bolivia	South America	Beetle
RS5271	Amboro Park, Bolivia	South America	Beetle
RS5275	Amboro Park, Bolivia	South America	Beetle
RS5264	Amboro Park, Bolivia	South America	Beetle
RS5200	Maduban, India	South Asia	Soil

### Determination of the rate of non-disjunction of the X chromosome

For each of 23 inbred strains of *P. pacificus*, 20 virgin, J4 stage hermaphrodites were placed on several 100-mm plates seeded with *E. coli *OP50. Worms were allowed to mature and lay eggs at 20°C. Once the worms hatched and were old enough to be sexed, males were counted and removed from each plate. To facilitate counting, the hermaphrodites left on the plate were killed by incubation at 45°C for 30 minutes. Their position in the plate was marked by dotting a sheet of plastic transparency affixed to the bottom of the plate using a fine tipped permanent marker. The transparency was scanned into a computer and dots were counted using the software package ImageJ with the cell counter plug-in. Typically between 300 and 2000 worms were counted for each plate and at least 7,700 progeny were counted for each strain. The same number of worms was counted for a smaller subset of strains to calculate rates of non-disjunction at 15°C and 25°C. The rate of non-disjunction was determined by counting the number of males relative to the total number of individuals in each of the selfing *P. pacificus *inbred strains. The error for the non-disjunction rates at 20°C was calculated assuming a Poisson distribution.

### Embryonic mortality

To determine the percentage of embryonic mortality, 10 J4 stage virgin hermaphrodites of each strain were transferred to single plates. These hermaphrodites were allowed to mature, lay eggs at 20°C and moved to a new plate every day until none of their eggs hatched for two consecutive days. Eggs were counted with the aid of a grid immediately after each hermaphrodite was transferred to a new plate. The embryo mortality was calculated by dividing the number of hatched worms by the number of eggs, and subtracting this ratio from 1.

### Mating assays

Male mating ability was assayed by placing three males along with one J4 stage virgin hermaphrodite in a 1.5 cm *E. coli *OP50 lawn on an agar plate. After 24 hours at 20°C, males were removed. Hermaphrodites were moved to a new plate every day and allowed to lay eggs. Worms of hatched eggs were counted and sexed once they reached maturity. Male mating ability was calculated by dividing the number of male progeny by the total number of progeny (males + hermaphrodites). We performed 10 replicates for each strain. The majority of the male progeny were sired by the parental males.

### Inbreeding depression

The hermaphrodites to be used in each cross (RS5207, RS5134 and RS5202) were first allowed to deplete their sperm supply. This was accomplished by transferring each hermaphrodite to a new plate every day until none of the eggs it laid hatched for two consecutive days. The hermaphrodite was then placed on a plate spotted with a small lawn (~1.5 cm) of OP50 with five males of the other strain to be utilized in the cross. Males of the strains RS5202 and JU723 were used and allowed to mate for 24 hours. Subsequently, males were removed and the hermaphrodites were transferred to new plates. The mated hermaphrodites were moved every day until none of their eggs hatched for two consecutive days. During this time period, the progeny from each individual were counted every day, and 50 random F1 individuals were selected and placed each onto their own plate. The selected F1 worms were allowed to mature, and as soon as they began laying eggs, they were transferred to a new plate every day until they yielded no progeny for two consecutive days. Data for brood size had to be square-root-transformed to normalize the residuals. Heterosis, defined as the relative increase in fitness of hybrids, was calculated according to Dolgin et al (2007) [33]. Negative values for heterosis indicate outbreeding depression. The means within the same cross were compared using Student *t*-tests.

### Sperm competition

Crosses were performed on agar in 60-mm Petri dishes spotted with a lawn of *Escherichia coli *strain OP50 (~1.5 cm in diameter) as food source [40], and kept at 20°C. Three Wild-type *P. pacificus *males were placed for three hours on the bacterial lawn along with a morphologically marked hermaphrodite. The morphological marker was a recessive mutant that produces a dumpy phenotype. Hermaphrodites of the strain PS312 *pdl-9 V *(*sy372*) [41] were mated and transferred to fresh plates at daily intervals until they stopped laying eggs. Eggs were allowed to hatch and worms were grown until body morphology and/or gender could be scored. Cross-progeny could be readily identified because of their Wild-type body morphology. This procedure was performed with 35 replicates.

The percentage graph was generated by first calculating the mean of the counts of each phenotype (dumpy, Wild-type hermaphrodite, Wild-type male) generated each day. The means for each of the three phenotypes were then summed to determine the total number of worms generated on each day. The percentages were then calculated by dividing each of the phenotype counts by the total progeny for that day and multiplying by 100.

## Results

**Figure 1:**
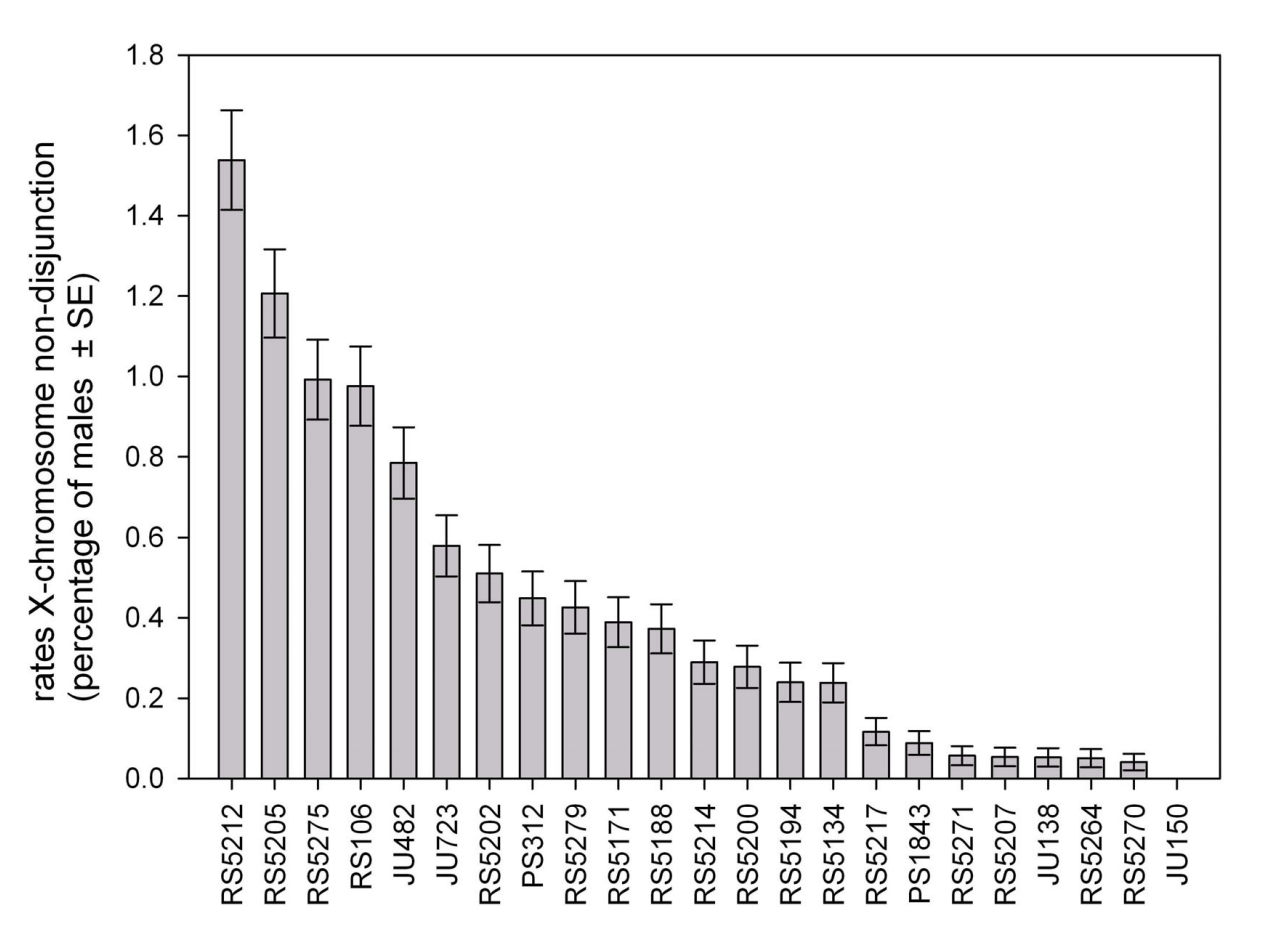
**Percentage of males in selfing *P. pacificus *inbred strains at 20°C**. The percentage of males was calculated from about 8,000 selfing progeny for each strain. Error bars indicate standard errors assuming a Poisson distribution.

In *C. elegans*, the frequency of XX hermaphrodites and XO males in a population is mainly driven by (1) the rate of non-disjunction events of the X chromosome during gametogenesis in hermaphrodites and (2) the ability of males to produce cross-progeny. *P. pacificus *also has an XX:XO sex determination system, where males occur at a frequency of 0.1% in standard laboratory cultures [42,43]. To determine how each of the above mentioned factors contributes to the frequency of males in *P. pacificus*, genetic variation of male production was determined for inbred lines isolated from various parts of the world (Table 1). On average, *P. pacificus *male frequency resulting from X chromosome non-disjunction among the 23 lines is 0.42% ± 0.05% SE. One strain (JU150) did not produce any males out of more than 8,000 self progeny. Of the strains that produced males, their percentages relative to total number of progeny ranged between 0.04% (RS5270) and 1.60% (RS5212) (Fig. 1). Rates of non-disjunction of *P. pacificus *are similar to *C. elegans *[0.33% ± 0.015% SD (n = 26)], where the lowest and highest non-disjunction rates differ by two orders of magnitude [32]. We compared *C. elegans *and *P. pacificus *overall differences in means using a *Z*-test with hypothesized variants based on observed binomial standard deviation. No statistical difference was detected in rates of X chromosome non-disjunction between these two androdioecious nematodes (*P *= 0.98).

**Figure 2:**
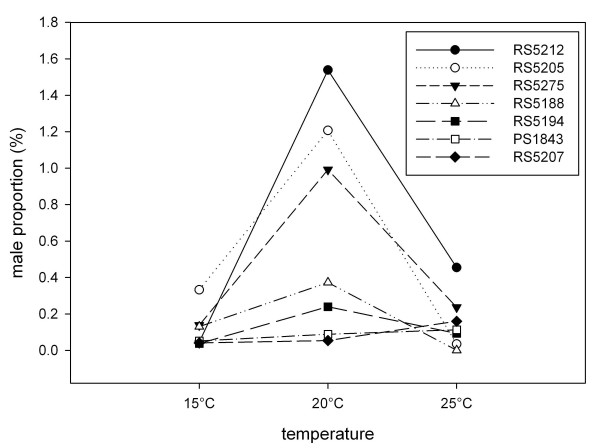
**The effect of temperature on the percentage of males in selfing inbred strains of *P. pacificus *at 15°C, 20°C and 25°C**. Non-disjunction rates were calculated by scoring the proportion of males from about 8,000 selfing progeny for each strain. Selfing progeny was counted from 5–10 replicate plates seeded with 20 parental worms each. Only mean values are shown in the graph for clarity.

In *C. elegans*, temperature has an obvious effect on the rate of X chromosome non-disjunction [32]. To determine if this was also the case for *P. pacificus*, we allowed a subset of strains to self at two additional temperatures, 15°C and 25°C. Strains were selected according to their rates of non-disjunction at 20°C and geographical location from where they were collected. It is clear that the highest proportion of males is produced at intermediate temperatures (Fig. 2). Strains isolated from geographical locations with similar climatic conditions presented very distinct non-disjunction rates (eg, Japanese strains RS5212 and RS5207) at 20°C, but more similar at lower temperatures. To determine whether there is interaction between environmental and genetic effects, an ANOVA test was performed (Table 2). The results indicate that there is a significant interaction between strain and temperature, which preclude the interpretation of the among strain and temperature differences.

**Table 2:** Analysis of variance for X chromosome non-disjunction (arcsine-squared transformed) in seven inbred *P. pacificus *strains in three temperatures.

Source	df	MS	*F*-Ratio	*P*
Strain	6	0.396	257.093	<0.0001
Temperature	2	0.550	357.477	<0.0001
Strain × temperature	12	0.422	274.119	<0.0001
Error	164	0.002		

Degrees of freedom are indicated (df) and mean squares (MS) are based on type III sum-of-squares.

**Figure 3:**
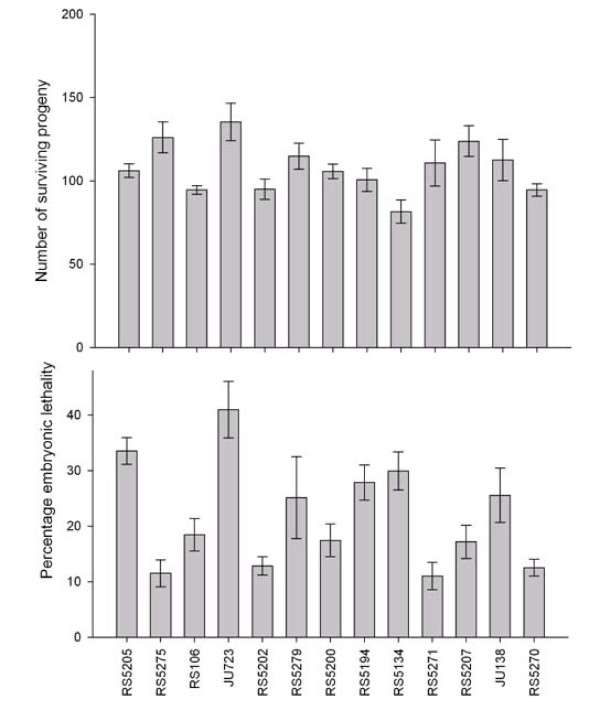
**Mean brood size (top) and embryonic mortality (bottom) for selfing inbred hermaphrodites of various *P. pacificus *strains**. Error bars indicate SEM. Strains are organized according to rates of X-chromosome non-disjunction at 20°C, from the highest to the lowest.

Rates of non-disjunction could affect brood sizes, since the production of each male generated by non-disjunction results in the production of one unviable or unfertile XXX hermaphrodite [44]. We selected 13 strains that span the range of X-chromosome non-disjunction rates (Fig. 3). The mean brood size of these inbred strains is 107 ± 15 SD progeny/hermaphrodite. There are significant genetic differences in brood size (F_12,100 _= 2.20, *P *< 0.05) and embryonic mortality (F_12,98 _= 5.72, *P *< 0.001) among different strains. However, no correlation was found between non-disjunction and embryonic mortality (*r *= 0.19, *P *= 0.52), or between non-disjunction and brood size (*r *= 0.10, *P *= 0.73). We also found no correlation between embryonic mortality and brood size (*r *= 0.17, *P *= 0.58).

**Figure 4:**
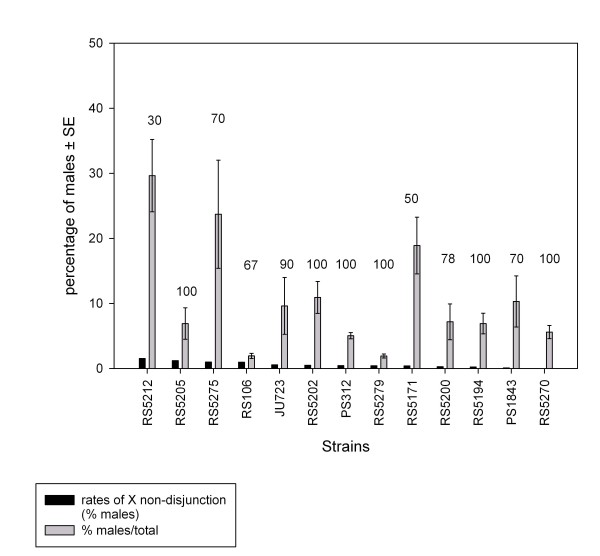
**Mating ability of *P. pacificus *strains**. Strains are ordered according to the rates of X chromosome non-disjunction at 20°C (black bars), from highest to lowest. Shaded bars represent the proportion of males in the progeny of successful crosses. Numbers above bars indicate the percentage of crosses that resulted in cross-progeny out of 10 replicates (except for RS106 and RS5200, which had 9 replicates). Error bars indicate standard errors.

In *C. elegans*, males of some strains are significantly more effective in mating than others [32,45,46]. We detected genetic variation in male fertilization ability among *P. pacificus *inbred lines as well (Fig. 4). Male fertilization ability was measured at two levels. First, we quantified male mating ability by scoring the proportion of matings that resulted in cross-progeny. Successful fertilization rates were equal to or higher than 90% for about half of the strains used for crosses. As an example of poor mating performance, males of strain RS5212 produced cross progeny with only 30% of the mated hermaphrodites. Second, we measured male fecundity by determining the proportion of male cross-progeny that resulted from successful fertilization events. *P. pacificus *showed significant variation of fecundity among strains (F_11,89 _= 4.13, *P *< 0.001). RS5212 males produced some of the highest proportions (30%) of male cross-progeny among the strains analyzed, indicative of high fecundity. Most of the other strains (10/13) produced 10% or less male cross-progeny. We could not find any correlation between rates of X-chromosome non-disjunction and male mating ability (*r *= -0.47, *P *= 0.105), or between X-chromosome non-disjunction and male fecundity (*r *= 0.59, *P *= 0.08).

**Figure 5:**
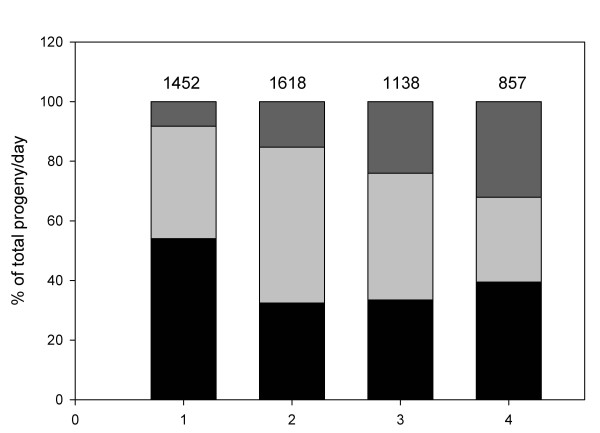
**Proportion of cross- and self-progeny in crosses between PS312 *pdl-9 *males and hermaphrodites**. Proportion of each phenotype for progeny per day. Self-progeny (dumpy): black; hermaphrodite cross-progeny: light grey; male cross-progeny: dark grey.

One of the factors that might influence efficient fertilization by males and sex ratios in cross progeny is sperm competition. Two types of sperm competition have been observed in nematodes: (1) between male and hermaphroditic sperm; (2) between X-bearing male sperm and male nullo-sperm. In *C. elegans *and *C. briggsae*, male sperm can efficiently outcompete hermaphroditic sperm [47-49], while in *C. briggsae *male X-bearing sperm has precedence over male nullo-sperm [49]. Precedence of X-bearing sperm results in bias towards hermaphroditic cross-progeny in the first days after mating. To identify cross-progeny and clarify the ratio of each sex after cross-fertilization in *P. pacificus*, we used a morphologically marked strain (see Materials & Methods). Hermaphrodites from strain PS312 *pdl-9 *(*sy372*), when mated to males, produced cross-progeny for about 4 days. Cross-progeny percentages peaked around the second to third day, accounting for approximately 65% of the brood (Fig. 5). Most animals died prematurely due to problems in egg-laying around the fifth day. Premature death may have precluded the usage of all male sperm stored in the hermaphrodite spermatheca and therefore the total proportion of male and hermaphrodite cross-progeny differed by 50% (X^2^_1 _= 11.42; P > 0.05). It is clear, however, that X-bearing sperm of PS312 males has precedence over nullo-sperm: hermaphrodites are produced in higher proportions than male cross-progeny in the first three days (Fig. 5). For PS312 there is approximately equal chance of either hermaphroditic sperm or male sperm to fertilize an oocyte.

In the short term, high levels of inbreeding increase the chances for accumulation of detrimental recessive mutations [50-52]. The combination of high mutation rates and high level of inbreeding can potentially cause species extinction, making self-fertile organisms evolutionary "dead ends" [50,51]. Therefore, some self-fertilizing animals maintain high proportions of males in their populations because they show substantial inbreeding depression [53,54]. *C. elegans*, however, has no inbreeding depression for life span, brood size and various other life-history traits [29,33,55,56].

**Figure 6:**
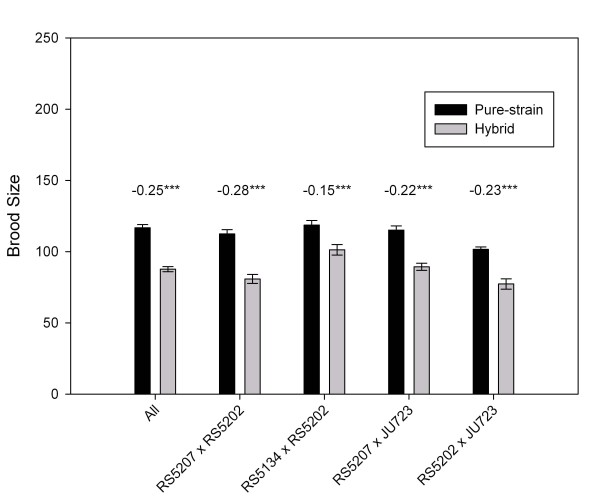
**Mean pure-strain (black bars) and hybrid (shaded bars) brood sizes**. Mean pure-strain is the midparent value. Numbers above the bars indicate heterosis values. Asterisks indicate significance of difference between pure-strains and hybrids in a Student's *t *test. ***P < 0.001.

To determine if *P. pacificus *has inbreeding depression, we made four sets of crosses between different strains and determined the brood sizes of the hybrid F1s (Fig. 6). We selected four strains based on the criterion of being genetically divergent from each other, as determined by phylogenetic analysis of mitochondrial DNA sequences [37](Sommer, unpublished). Similar to *C. elegans*, we detected outbreeding depression, where the *P. pacificus *pure strains outperformed the hybrids [33]. Paired comparisons consistently resulted in negative heterosis values, which reflected the smaller brood sizes of hybrids when compared to the parental pure strains (Fig. 6).

## Discussion

Given that males in androdioecious species are not essential for reproduction, several hypotheses have been proposed for their persistence in some nematode species. Androdioecy is thought to be an intermediate mating system that would be stable only in very specific conditions [57,58], including high inbreeding depression and high male mating efficiency [18,30]. Chasnov and Chow [29] suggested that *C. elegans *males are still maintained because mating is frequent enough to prevent degeneration of male-specific genes by mutations. Extremely low levels of outcrossing and high rates of selfing can result in accumulation of deleterious mutations, which cause extinction [19,51]. The evolutionary forces responsible for the maintenance of males in *C. elegans *and the time scale for their persistence are not yet very well understood.

To understand how significant the role of males is in distant relatives of *C. elegans*, we measured variation of various life-history traits important for outcrossing in strains of the nematode *P. pacificus*. The genus *Pristionchus *has at least four species that evolved androdioecy independently, including *P. pacificus *[39]. Although *P. pacificus *and *C. elegans *are distant relatives and have many developmental differences [26], they share some common features: the same type of sex determination (XX:XO), males are relatively rare [42], and the hermaphrodite is a modified female whose gonad first produces sperm and then shifts to produce oocytes [59]. Our results show that 23 *P. pacificus *strains produce males by X chromosome non-disjunction at comparable rates to *C. elegans*.

Production of males by non-disjunction comes at a cost, because for every XO male generated one XXX unviable hermaphrodite is produced. However, we did not find a correlation between high non-disjunction rates with lower brood sizes or higher rates of embryonic mortality. The percentage of embryonic mortality seems high for most strains, typically ranging between 10–30%. Similar rates were found for other selfing nematodes [60], but the causes for high mortality are unknown. It is possible that our results are slight overestimates of embryonic mortality, because we could not rule out the possibility that some hatched larvae died at the wall or edge of the plate before being scored. However, we repeated the assays and confirmed that strains scored as having high lethality displayed by many dead embryos.

Brood sizes in *P. pacificus *are about half of those of *C. elegans *[46,61] (this report). Although the difference might be partially explained by the higher embryonic lethality in *P. pacificus*, additional factors could also play a role. *P. pacificus *might be producing fewer sperm as an adaptation for earlier reproduction. Because spermatogenesis precedes oogenesis, the fewer sperm are produced, and the hermaphrodite can start reproducing earlier. *C. elegans *mutants that increase sperm number, for instance, were shown to be of disadvantage under natural conditions because the additional spermatogenesis delays oogenesis and therefore the minimum generation time [62]. Further work would be necessary to test whether the smaller brood size in *P. pacificus *is due to lesser sperm production.

Male mating is one of the most complex behaviors in nematodes [63,64], and many factors can influence successful mating: male sexual drive, ability of the male to locate the hermaphrodite, ability of the male to insert its spicules into the vulva, and amount of sperm transferred in each mating. Mating assays are typically performed in agar plates, where hermaphrodites and males are placed in a small spot of bacteria for a few hours or days [65,29,32,68]. We performed two tests for mating ability. The quantitative assay determined the proportion of hermaphrodites fertilized by 1–3 males in a 24-hour period. The qualitative assay tested male fecundity once they mated with hermaphrodites. Our results show that most hermaphrodites are fertilized by males in the above conditions. However, there is some variability of the number of progeny sired by each male. Some strains are clearly more fecund, such as RS5212 and RS5275. Because there is no standard nematode mating assay, it is not possible to compare our results with the ones performed in *C. elegans*. Given the benign conditions in which the crosses were performed, it is very likely that in more natural conditions male mating ability would be much lower. Further studies are required to access the effects of male nutritional status [69], habitat surface [70], health status [71] and longevity [72] on mating success.

The higher competitive ability of the male sperm to outperform the hermaphroditic sperm in fertilizing oocytes would be an indication for selection acting on males. This is well documented for *C. elegans *strain N2 [47], where mated hermaphrodites produce outcross progeny almost exclusively in the first 24 hours after mating. *C. elegans *male sperm can anchor to the wall of the spermatheca, preventing them from being swept away by the exiting eggs. In our study with *P. pacificus *PS312, we observed that male sperm is equally likely to fertilize an oocyte as a hermaphrodite sperm (Fig. 5). It is possible that *P. pacificus *male sperm lost the ability to anchor themselves, or that the shape of the spermatheca does not favor attachment. In fact, it has been observed that the shape of the *P. pacificus *spermatheca is very different from the one in *C. elegans *[73]. In *C. elegans*, the larger size of the male sperm has been reported to give a higher competitive advantage due to their faster crawling and ability to displace smaller sperm [74,75]. It remains to be determined whether the size of the sperm also plays a role in *P. pacificus*.

Differences in the competitive ability of *P. pacificus *X-bearing sperm over nullo-sperm were detected. Precedence in X-bearing sperm provides an advantage to faster reproduction and ability to colonize new habitats [49]. *P. pacificus*, which feeds on bacteria and fungi that grow on insect carcasses, must clearly rely on early progeny that are self-fertile to grow on such ephemeral habitats [76].

Theoretical models predict that males persist in androdioecious species with high levels of inbreeding depression [18,30]. Our results suggest that *P. pacificus *has outbreeding depression, where the hybrid has lower fitness than the pure strains. This might be caused by hybrid breakdown of co-adapted gene complexes, as found for *C. elegans *[33]. It should be noted, however, that we measured only one fitness trait in our assays for inbreeding depression (brood size). Different results could be produced for other ecologically-relevant traits, such as longevity, pathogen susceptibility [77], and ability to undergo dauer formation [78]. Other selfing species, such as *C. briggsae*, do not show outbreeding depression [79]. This indicates that this phenomenon is not particular to self-fertilizing nematodes.

The low rates of male production by hermaphrodite selfing, low fecundity rates of males, poor male sperm competition and outbreeding depression suggest that *P. pacificus *males are being selected against. This could explain the low level of recombination observed between *P. pacificus *strains, indicating low outcrossing rates [37]. Persistence of males in *P. pacificus *could be explained as a byproduct of the sex determination system, where males are generated by X chromosome non-disjunction events in hermaphrodite meiosis. It would be useful to study other kinds of evidence for selection acting on males, such as production of pheromones by hermaphrodites [80-82], or production of substances by the males that induce immobilization of hermaphrodites during copulation [68].

## Conclusion

In summary, this study shows that many life-history traits of *P. pacificus *are similar to *C. elegans*, such as spectrum of non-disjunction rates, influence of temperature in non-disjunction, generally poor male mating ability and outbreeding depression. In contrast, *P. pacificus *has lower brood sizes and poor male sperm competiveness. Additional studies would be necessary to determine whether males in androdioecious species can be evolutionarily stable.

## Authors' contributions

SK inbred the strains for 10 generations and provided preliminary data on rates of non-disjunction and brood size. AC, CS and APS participated in experimental design, data collection, data analysis and writing of the manuscript. AC and APS coordinated the project and supervised the practical work. All authors read and approved the final manuscript.
